# Liver-specific metastases as an independent prognostic factor in cancer patients receiving hospice care in hospital

**DOI:** 10.1186/s12904-023-01180-x

**Published:** 2023-05-24

**Authors:** Kun-Siang Huang, Yun-Hwa Huang, Chao-Tung Chen, Chia-Pei Chou, Bo-Lin Pan, Chih-Hung Lee

**Affiliations:** 1 Department of Family Medicine, Kaohsiung Chang Gung Memorial Hospital, Kaohsiung, Taiwan; 2 Department of Dermatology, Kaohsiung Chang Gung Memorial Hospital, Kaohsiung, Taiwan; 3 Chang Gung University College of Medicine, Taoyuan, Taiwan; 4 Institute of Translational Research in Biomedicine, Kaohsiung Chang Gung Memorial Hospital, Kaohsiung, Taiwan; 5 National Sun Yat-Sen University College of Medicine, Kaohsiung, Taiwan

**Keywords:** Hospice, Survival, Liver metastasis, Palliative, Enteral nutrition

## Abstract

**Background:**

Survival prediction is important in cancer patients receiving hospice care. Palliative prognostic index (PPI) and palliative prognostic (PaP) scores have been used to predict survival in cancer patients. However, cancer primary site with metastatic status, enteral feeding tubes, Foley catheter, tracheostomy, and treatment interventions are not considered in aforementioned tools. The study aimed to investigate the cancer features and potential clinical factors other than PPI and PaP to predict patient survival.

**Methods:**

We conducted a retrospective study for cancer patients admitted to a hospice ward between January 2021 and December 2021. We examined the correlation of PPI and PaP scores with survival time since hospice ward admission. Multiple linear regression was used to test the potential clinical factors other than PPI and PaP for predicting survival.

**Results:**

A total of 160 patients were enrolled. The correlation coefficients for PPI and PaP scores with survival time were -0.305 and -0.352 (both *p* < 0.001), but the predictabilities were only marginal at 0.087 and 0.118, respectively. In multiple regression, liver metastasis was an independent poor prognostic factor as adjusted by PPI (β = -8.495, *p* = 0.013) or PaP score (β = -7.139, *p* = 0.034), while feeding gastrostomy or jejunostomy were found to prolong survival as adjusted by PPI (β = 24.461, *p* < 0.001) or PaP score (β = 27.419, *p* < 0.001).

**Conclusions:**

Association between PPI and PaP with patient survival in cancer patients at their terminal stages is low. The presence of liver metastases is a poor survival factor independent of PPI and PaP score.

## Background

Hospice care provides symptom-focused end-of-life care for patients with terminal cancer or other diseases of terminal status and improves quality of life for these patients and their families [1, 2]. Notably, cancer patients have a higher hospice care utilization rate than non-cancer patients (15.84%, and 1.04%, respectively) [3]. Hospice care can be delivered either in the community or hospital setting. Evidence suggests that hospital-based palliative care by specialist provides better benefit for quality of life and symptom burden in terminally ill patients [4]. In Taiwan, hospice care in the ward setting consists of a professional palliative care team for holistic care to terminal illness patients in a specialized ward.

Prognostication in advanced cancer patients is crucial because the clinical decisions and treatments may be determined depending on the life expectancy [5]. In hospice wards, specialists optimize quality of life of the patients by palliative treatment. For those patients with anticipated prolonged survival, certain procedures and treatments may be considered, but for those with more limited survival, patients and care teams may favor avoiding these interventions [6]. Therefore, many prognostic models have been developed to predict survival of cancer patients in terminal stages. The Palliative Prognostic Index (PPI) is widely used for survival prediction in terminally ill cancer patients based on palliative performance scale, oral intake, edema, dyspnea at rest, and delirium. The PPI score ranges from 0 to 15. The PPI score over 6 indicates the predicted survival time less than 3 weeks with a sensitivity of 80% and a specificity of 85%; the PPI score over 4 indicates the predicted survival time less than 6 weeks with a sensitivity of 80% and a specificity of 77% [7]. Another prognostic model, the Palliative Prognostic (PaP) score, has also been used to predict the 30-day-survival probability using variables including Clinical Prediction of Survival, Karnofsky Performance Status, anorexia, dyspnea, total white blood count, and lymphocyte percentage. The PaP score ranged from 0 to 17.5 and is categorized into three groups (Group A < 5.5; Group B 5.6–11; Group C > 11.1). The 30-day-survival probability is over 70% in Group A, 30% to 70% in Group B, and less than 30% in Group C [8].

Several studies have validated the survival prediction of the PPI and PaP scores in terminal cancer patients with good survival discrimination between the different groups [9]. Stone et al. validated the PPI score in hospitalized and home care patients, and the positive predictive values were 91% and 86% with the score cutting value at 4 and 6 respectively [10]. Maltoni at el. validated PaP score via a multicenter study evaluating in-hospital cancer patients at their terminal stage (*n* = 451). PaP scores were significantly associated with patient survival (log rank = 203.8, *P* < 0.0001) [11]. Another PaP score validation study focusing on hospital-based palliative medicine consultation service showed significant difference among the 3 risk groups [12]. These studies determined the survival time by categorizing the patients into different groups according to PPI and PaP scores, but the linear association of PPI and PaP scores with survival time were unknown.

Though the PPI and PaP score are widely used and validated for advanced cancer patients, there remain several limitations within the two prediction models. PPI and PaP score depend on mostly subjective symptom and functional performance assessment. For the PaP score, the clinical prediction of survival accounts for substantial proportion of total scores, which may be subjective and inconsistent among different physicians. Prior work has indicated that high C-reactive protein, low albumin, and high neutrophil-to-lymphocyte ration are poor prognostic factors [13], but there are no laboratory values contained within the PPI. Other work supports use of primary cancer site and status of metastatic disease in survival prediction, neither of which are taken into consideration by the PPI or PaP score. Gwilliam et al. developed prognostic prediction tools in advanced cancer patients, and they found primary cancer site (breast cancer and male genital cancer), presence of distant metastases, and metastases sites to liver or bone would also help in predicting survival [14]. Chow et al. also proposed a survival predictive model that consisted of non-breast cancer, metastases other than bone, and Karnofsky performance scale < 60 in metastatic cancer patients (lung, breast and prostate cancers were the most common primary cancer sites) [15].

In addition, some medical treatments or interventions, such as artificial nutrition (parenteral nutrition or enteral tube feeding), antibiotic use, palliative total sedation, blood transfusions, and opioid use for end-of-life symptom management may influence survival time [15, 16]. Oxygen therapy is often used for breathlessness in palliative care but with uncertain efficacy and impact on survival [17]. Tracheostomy provides patent airway for terminal cancer patients, but its effect on survival is unknown. Foley catheters are used in patients who have lost their self-voiding ability in certain hospice care settings, which may indicate terminal illness progression. These treatments are not included in prior prognostic prediction models.

Therefore, the goal of this study was to evaluate the linear association of PPI and PaP scores with survival time. This study aimed also to explore other possible clinical features (in particular the primary sites of cancer and metastatic sites) and treatments (in particular the enteral feeding tubes) not included in PPI or PaP score associated with survival time in cancer patients admitted to hospice ward.

## Method and material

### Study design and patient selection

We conducted a retrospective study and identified cancer patients at their terminal stages who were admitted to the Kaohsiung Chang Gung Memorial Hospital hospice ward between 1^st^, January 2021 and 31^st^, December 2021. We excluded patients who admitted to hospice ward due to non-cancer terminal diagnosis and patients whose death date could not be obtained. We also excluded the patients whose blood test time was over 14 days before hospice ward admission because the white blood cell count and lymphocyte percentage are needed in the PaP scoring process. Two separate family medicine physicians who specially trained in palliative and hospice care reviewed each patient’s electronic medical chart and records. We recorded patient demographic data, terminal disease diagnosis, laboratory findings, treatments during hospitalization, PPI, PaP score, and survival time since hospice ward admission for analysis. We made note of treatments started after admission to the hospice ward in order to explore these treatments’ impact on survival.

### Statistical analysis

We performed statistical analyses by SPSS version 25.0 software (IBM corp., Armonk, NY, USA). We used the simple linear regression to evaluate the correlation of survival time since hospice ward admission with PPI and PaP score. We conducted univariate analysis to examine the correlation of clinical factors and survival time since hospice ward admission. We performed multiple linear regression to explore clinical factors affecting the survival time since hospice ward admission with PPI and PaP score, respectively. For all analyses, P less than 0.05 was statistically significant.

### Ethics statements

The retrospective study was approved by Chang Gung Memorial Hospital Foundation Institutional Review Board(202201053B0) with waiver of written consents.

## Results

### Demographic data of 160 patients admitted to hospice ward

A total of 160 hospice ward admission cancer patients were included in this study. Among the participants, the mean age was 65.8 years old, and 86(54%) were male (Table 1). Regarding the primary cancer sites, hepatobiliary cancer accounted for 22.5%, urogenital cancer, head and neck cancer, and lung cancer accounted for 11.9% each, esophageal and gastric cancer totaled 11.3%, colorectal cancer accounted for 10.6%, and the breast cancer accounted for 7.5%. Among the patients, 80(50%) had lymph node metastases, 67(42.1%) had lung metastases, 63(39.4%) had liver metastases, 58(36.3%) had bone metastases, and 17(10.6%) had brain metastases. Regarding tubes and catheters, 64(40%) patients had Foley catheter, 38(23.8%) had nasogastric tube, 20(12.5%) had feeding gastrostomy or jejunostomy, and 16(10%) had tracheostomy. Regarding the medical treatment during hospice ward admission, 88(55%) patients received subcutaneous morphine, 6(3.8%) received blood transfusion, 4(2.5%) received total sedation, and 3(1.9%) received parenteral nutrition. The mean survival time since hospice ward admission was 16.8 days. The mean PPI and PaP score were 7.23 and 11.40 days, respectively.

**Table 1:** Demographic data of hospice ward admission patients

Variables	Hospice ward admission patients *N* = 160	
Sex		
Male (number, %)	86	54%
Female (number, %)	74	46%
Age (mean, SD)	65.8	11.5
Age > = 65 years old (number, %)	101	59.1%
Charlson comorbidity index (mean, SD)	9.54	2.35
Palliative prognostic index (mean, SD)	7.23	3.74
Palliative prognostic score (mean, SD)	11.40	4.74
Survival days since hospice ward admission (mean, SD)	16.8	23.7
Hospice ward hospitalization days (mean, SD)	10.5	14.1
Cancer Sites		
Hepatobiliary cancer (number, %)	36	22.5%
Urogenital system cancer (number, %)	19	11.9%
Breast cancer (number, %)	12	7.5%
Colorectal cancer (number, %)	17	10.6%
Lung cancer (number, %)	19	11.9%
Esophageal and Gastric cancer (number, %)	18	11.3%
Head and Neck cancer (number, %)	19	11.9%
Others (number, %)	20	12.5%
Cancer Metastases sites		
No distant metastases (number, %)	19	11.9%
1–2 metastases sites (number, %)	79	49.4%
More than 3 metastases sites (number, %)	62	38.8%
Brain metastases (number, %)	17	10.6%
Lymph node metastases (number, %)	80	50%
Lung metastases (number, %)	67	42.1%
Liver metastases (number, %)	63	39.4
Bone metastases (number, %)	58	36.3
Activity daily living < 20 when admission (number, %)	92	57.5%
Laboratory data		
Neutrophil percentage (mean, SD)	83.42	12.37
Lymphocyte percentage (mean, SD)	8.23	8.07
Neutrophil to lymphocyte ratio (mean, SD)	20.90	23.90
Nasogastric tube use (number, %)	38	23.8%
Feeding gastrostomy or jejunostomy (number, %)	20	12.5%
Tracheostomy (number, %)	16	10%
Foley catheter use (number, %)	64	40%
Treatments when starting hospice ward admission		
Parenteral nutrition (number, %)	6	3.8%
Antibiotic (number, %)	71	44.4%
Oxygen (number, %)	136	85%
Treatments during hospice ward hospitalization		
Subcutaneous morphine use (number, %)	88	55%
Total sedation (number, %)	4	2.5%
Blood transfusion (number, %)	6	3.8%
Parenteral nutrition (number, %)	3	1.9%

### Low level of association of both palliative prognostic index and palliative prognostic score with patient survival

**Fig. 1:**
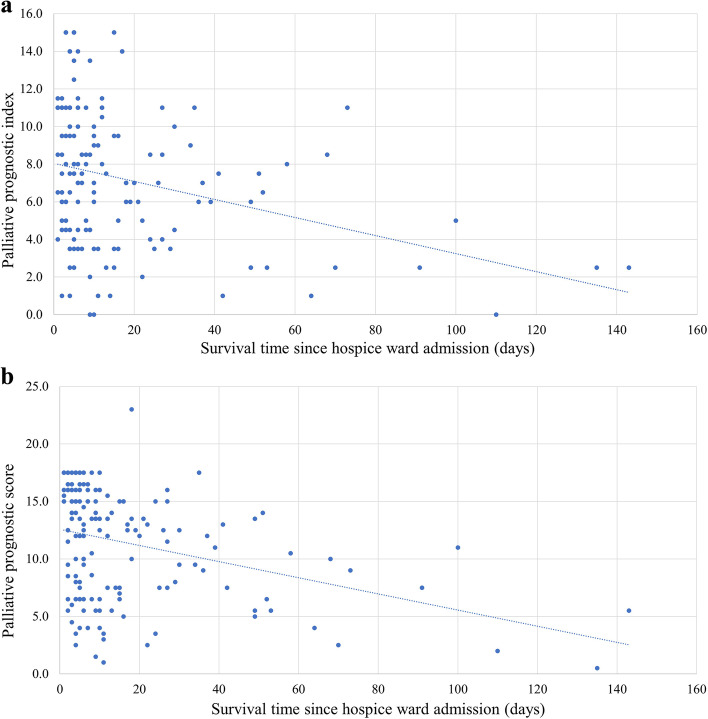
**a** The correlation of survival days and palliative prognostic index. Adjusted *R*^2^ = 0.087, β = -0.305, *p* value < 0.001. **b** The correlation of survival days and palliative prognostic score. Adjusted *R*.^2^ = 0.118, β = -0.352, *p* value < 0.001

To confirm that PPI and PaP score, both of which are known survival predictors in patients of hospice, could be applied in our study, we performed association analysis between them. Figure 1a and b revealed the correlation of survival days since hospice ward admission with PPI and PaP score, respectively. The correlation coefficients were -0.305 and -0.352 for PPI and PaP score. The adjusted R^2^ was only 0.087 and 0.118 with PPI and PaP score respectively, which indicated low prediction ability of the survival days for these two prognostic tools.

### Independent factors associated with poor patient survival

Since the PPI and PaP score were poor predictors for patient survival in bivariate association analysis in our study, we sought to explore other factors, independent of PPI and PaP score, that may be associated with patient survival. We first performed the univariate associated analysis to predict survival (Table 2). Head and neck cancer, feeding gastrostomy/jejunostomy tubes, tracheostomy, total sedation, and blood transfusion during hospice ward admission were associated with significantly prolonged survival time. Conversely, hepatobiliary cancer, liver metastases, higher neutrophil to lymphocyte ratio, and antibiotic use were negatively correlated with survival time.

**Table 2:** Univariate analysis of clinical factors associated with survival time since hospice ward admission

	Correlation coefficient	*P* value
Sex	-0.115	0.148
Age	0.001	0.987
Age > = 65 years old	0.069	0.386
Charlson comorbidity index	0.023	0.774
Palliative prognostic index	-0.305**	< 0.001
Palliative prognostic score	-0.352**	< 0.001
Hospice ward hospitalization days	0.710**	< 0.001
Cancer Sites		
Hepatobiliary cancer	-0.174*	0.028
Urogenital system cancer	-0.059	0.461
Breast cancer	-0.069	0.384
Colorectal cancer	-0.024	0.762
Lung cancer	-0.054	0.500
Esophageal and Gastric cancer	0.110	0.164
Head and Neck cancer	0.273*	< 0.001
Others	0.035	0.663
Cancer Metastases sites		
Brain metastases	0.058	0.465
Lymph node metastases	-0.027	0.738
Lung metastases	-0.020	0.799
Liver metastases	-0.228**	0.004
Bone metastases	-0.018	0.824
Activity daily living < 20 when admission	0.068	0.392
Laboratory data		
Neutrophil percentage	-0.109	0.169
Lymphocyte percentage	0.078	0.329
Neutrophil to lymphocyte ratio	-0.170*	0.032
Nasogastric tube use	0.069	0.385
Feeding gastrostomy or jejunostomy	0.400**	< 0.001
Tracheostomy	0.342**	< 0.001
Foley catheter use	-0.057	0.476
Treatments when starting hospice ward admission		
Parenteral nutrition use	0.000	1
Antibiotic use	-0.169*	0.033
Oxygen use	-0.070	0.377
Treatments during hospice ward hospitalization		
Subcutaneous morphine use	-0.119	0.135
Total sedation	0.221**	0.005
Blood transfusion	0.327**	< 0.001
Parenteral nutrition	0.048	0.549

^*^p value < 0.05, ** *p* value < 0.01

### Liver specific metastasis as a poor survival factor independent of PPI and PaP

We performed multiple regression analysis based on the results of the univariate analysis in order to adjust for potential confounders and to investigate whether there were independent factors that predicted survival other than PPI and PaP score (Table 3). After adjustment for age, sex, and cancer metastasis sites, PPI had significant negative association with survival time (β = -1.058, *p* = 0.021). Liver metastases were also negatively associated with survival time (β = -8.495, *p* = 0.013). Feeding gastrostomy/jejunostomy tubes and blood transfusions during hospice ward admission had positive association with survival time, with β = 24.461 (*p* < 0.001) and β = 41.511 (*p* < 0.001), respectively. The adjusted R^2^ in this multiple linear regression was 0.338 (*p* < 0.001).

**Table 3:** The multiple linear regression of palliative prognostic index with other clinical factors and survival time

	β	β 95% CI	*P* value
Palliative prognostic index	-1.058*	-1.995, -0.161	0.021
Sex	0.277	-6.406, 6.959	0.935
Age	0.225	-0.064, 0.513	0.126
Brain metastases	3.450	-6.887, 13.786	0.511
Lymph node metastases	-2.193	-8.715, 4.330	0.508
Lung metastases	0.723	-5.785, 7.231	0.826
Liver metastases	-8.495*	-15.195, -1.795	0.013
Bone metastases	3.315	-3.447, 10.076	0.334
Feeding jejunostomy or gastrostomy	24.461**	13.189, 35.733	< 0.001
Blood transfusion during hospice ward hospitalization	41.511**	24.756, 58.266	< 0.001
Adjusted R^2^	0.338**		< 0.001

*CI* Confidence Interval

^*^p value < 0.05, ** *p* value < 0.01

The multiple linear regression based on PaP score associated with survival time was shown in Table 4. After adjustment for age, sex, and cancer metastasis sites, the PaP score was negatively associated with survival time (β = -1.192, *p* = 0.001), and liver metastases also were negatively associated with survival time (β = -7.139, *p* = 0.034). Nasogastric tube use, feeding jejunostomy or gastrostomy, and blood transfusion during hospice ward hospitalization were positively associated with survival time, and the β were 7.898(*p* = 0.044), 27.419(*p* < 0.001), 36.873(*p* < 0.001), respectively. The adjusted R^2^ in this model was 0.375(*p* = < 0.01).

**Table 4:** The multiple linear regression of palliative prognostic score with other clinical factors and survival time

	β	β 95% CI	*P* value
Palliative prognostic score	-1.192**	-1.876, -0.508	0.001
Sex	1.652	-4.961, 8.264	0.622
Age	0.234	-0.047, 0.516	0.102
Brain metastases	2.072	-8.176, 12.320	0.690
Lymph node metastases	-1.830	-8.204, 4.544	0.571
Lung metastases	0.699	-5.630, 7.027	0.828
Liver metastases	-7.139*	-13.734, -0.545	0.034
Bone metastases	2.790	-3.831, 9.411	0.406
Nasogastric tube use	7.898*	0.209, 15.586	0.044
Feeding jejunostomy or gastrostomy	27.419**	16.403, 38.435	< 0.001
Blood transfusion during hospice ward hospitalization	36.873**	20.220, 53.526	< 0.001
Adjusted R^2^	0.375*		0.044

*CI* Confidence Interval

^*^p value < 0.05, ** *p* value < 0.01

### Liver metastasis but not brain, bone, lung, and lymph node metastasis, is an independent poor survival factor in the cancer patients receiving hospice care

**Fig. 2:**
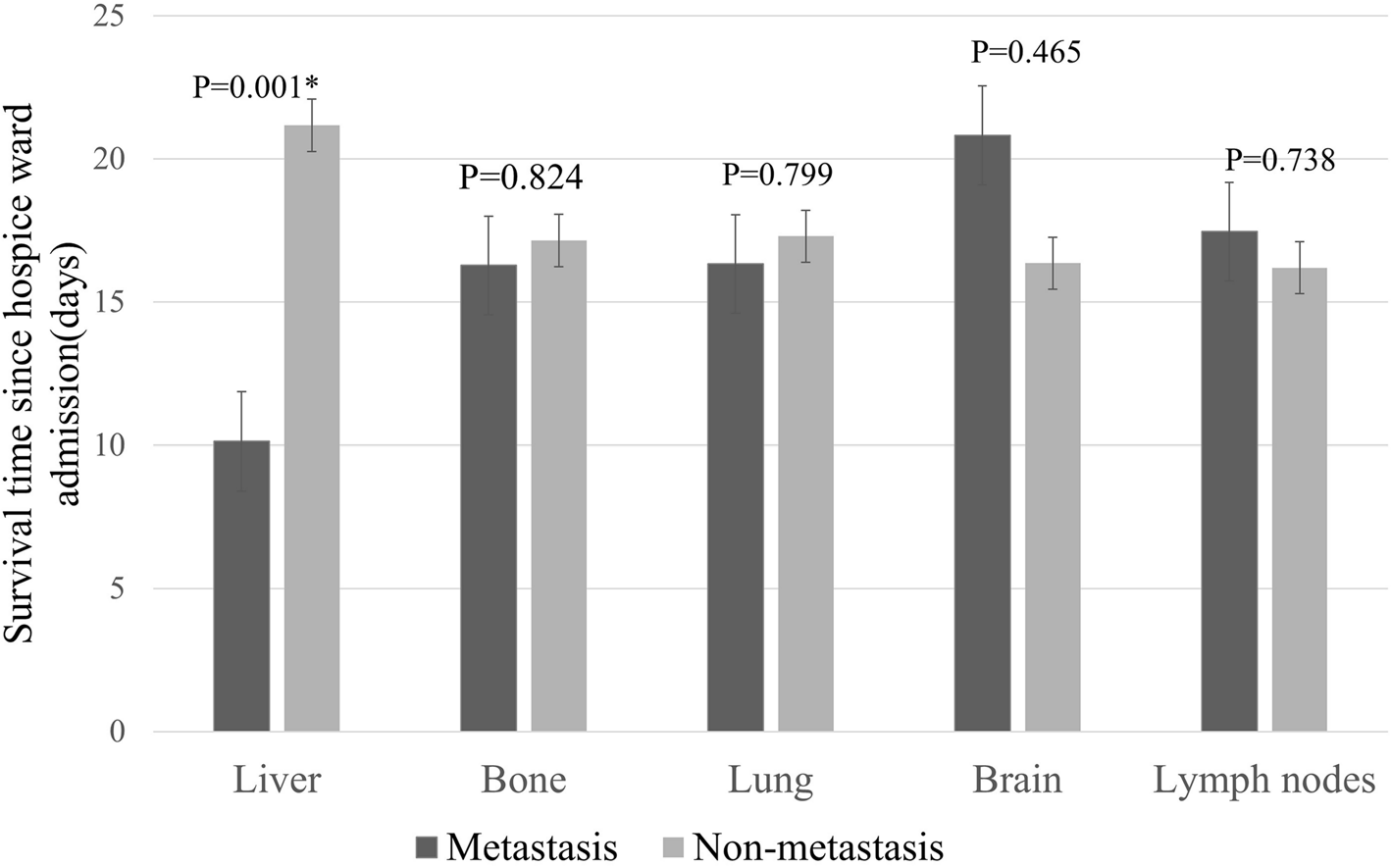
The survival time among different metastases sites. Independent T test was performed to examine the survival time among patients with and without different organ metastases. *p < 0.05

Since both regression models showed the common risk of liver metastasis in decreased survival, we charted the data to show the survival time with different type of metastases, including liver, bone, lung, brain, and lymph nodes (Fig. 2). The data indicated increased risk of shortened survival specific to liver metastasis in these cancer patients receiving hospice ward care.

## Discussion

From the analysis of our data, PPI and PaP scores were negatively associated with survival time in cancer patients admitted to hospice ward but with limited predictability. In cancer patients admitting to hospice ward, liver metastasis was found to be a poor prognostic factor among other metastases sites. In addition, feeding jejunostomy or gastrostomy and nasogastric tube use may prolong limited survival days in these terminal cancer patients.

This is the first study indicating that liver metastasis was a poor prognosis factor based on PPI and PaP score in cancer patients admitted to hospice ward. In our study, the presence of liver metastases was associated with reduced survival time of approximately seven to eight days. While our study was the first to describe this pattern in this specific patient population near end of life, our findings are consistent with other work in the literature. Vigano et al. reported that liver metastases were associated with poor survival in lung, breast, and gastrointestinal cancer patients (hazard ratio 2.2 and 2.4 in different models) [18]. In Vigano’s study, the mean survival of included advanced cancer patients was 15.3 weeks, whereas the mean survival time in our study was 16.8 days. Bilen et al. reported that the overall survival was shorter in cancer patients receiving immunotherapy with liver metastases than those without liver metastases (8.1 vs 21.9 months, *p* = 0.0048) [19]. Wang et al. also reported that patients with liver metastases at time of cancer diagnosis had median survival of four months [20]. The literature indicates that liver metastases are a poor prognostic factor throughout the disease spectrum, and our findings support this in patients at the terminal stage. The physiologic impact of liver metastases likely contributes to limited survival. The liver is a frequent site of metastases due to anatomical location and immunosuppressive environment [21]. Once affected by metastatic disease, metastatic tumor burden can impair normal physiologic function, possible leading to acute liver failure and subsequent jaundice, hepatic encephalopathy, and coagulopathy [22, 23] which may be associated with mortality in advanced cancer patients. Thus, the presence of liver metastasis in hospice care provided clinicians the clue for poor prognosis.

Regarding the presence of feeding tube, we found that terminal cancer patients admitted to hospice ward with gastrostomy or jejunostomy had prolonged survival of 24 to 27 days. For patients with nasogastric tube, had prolonged survival time of 7.9 days. The prevalence of cachexia in cancer patients receiving palliative care was estimated to range from 12 to 85% by different definitions [24]. When the patients developed cachexia or difficulty in oral intake, enteral feeding could be exercised. While enteral feeding routes may provide nutrition when the digestion function has failed in terminal cancer patients, enteral feeding for patients end-stage disease remains controversial, especially when poor oral intake relates to overall disease progression [25]. The current study and studies from others showed that terminal cancer patients who receive enteral nutrition either in hospice ward or in home palliative care have prolonged survival [26, 27]. However, enteral feeling may be prone to complications such as breakdown of tube site, tube displacement, tube obstruction, general edema, and local infection [28]. The American Society of Clinical Oncology guideline even suggested enteral feeding should not be used routinely in advanced cancer patients with cachexia [29]. Given the complexity of the issue, shared decision-making for enteral nutrition is essential in patients with terminal cancer. Our study results may provide some useful data for clinicians engaging in shared decision making about enteral feeding in advanced cancer patients receiving hospice care.

There were several limitations in our study. First, this was a single center study with only 160 patients included. For some variables, such as blood transfusion during hospitalization, the case number was small, therefore, we did not discuss about the blood transfusion in our article. Second, although all the patients admitted to hospice ward were routinely assessed for PPI and PaP score, the scores were evaluated by different physicians, whose scoring may have some inconsistency. Third, the primary cancer site categorization and distribution may also influence our analysis results. Further prospective studies are needed to validate the PPI and PaP scores and assess the impact of liver metastasis and enteral feeding in survival prediction in advanced cancer patients.

## Conclusion

The PPI and PaP scores were negatively associated with survival time in cancer patients admitted to a hospice ward, though with limited predictability. When assessing prognosis via PPI and PaP scores, enteral feeding may be associated with prolonged survival time, and liver metastases represents an independent poor prognostic factor. Liver metastasis and enteral feeding may be taken into consideration when assessing prognosis in advanced cancer patients by PPI and PaP scores.

## Data Availability

Requests for data access should be addressed to the leading author (KSH) and will be reviewed and responded to.

## Abbreviations

PPI: Palliative Prognostic Index
PaP: Palliative Prognostic
